# 17. Comparative Assessment of a Machine Learning Model and Rectal Swab Surveillance to Predict Hospital Onset *Clostridioides difficile*

**DOI:** 10.1093/ofid/ofab466.017

**Published:** 2021-12-04

**Authors:** Erkin Ötleş, Jeeheh Oh, Alieysa Patel, Micah Keidan, Vincent B Young, Krishna Rao, Jenna Wiens

**Affiliations:** 1 University of Michigan, Ann Arbor, Michigan; 2 Department of Internal Medicine, Infectious Diseases Division University of Michigan, Ann Arbor, Michigan, Ann Arbor, MI

## Abstract

**Background:**

Hospital onset *Clostridioides difficile* infection (HO-CDI) is associated with significant morbidity and mortality. Screening individuals at risk could help limit transmission, however swab-based surveillance for HO-CDI is resource intensive. Applied to electronic health records (EHR) data, machine learning (ML) models present an efficient approach to assess patient risk. We compare the effectiveness of swab surveillance against daily risk estimates produced by a ML model in detecting patients who will develop HO-CDI.

**Methods:**

Patients presenting to Michigan Medicine’s ICUs and oncology wards between June 6th and October 8th 2020 had rectal swabs collected on admission, weekly, and at discharge from the unit, as part of VRE surveillance. We performed anaerobic culture on the residual media followed by a custom, multiplex PCR on isolates to identify toxigenic *C. difficile*. Risk of HO-CDI was calculated daily for each patient using a previously validated EHR-based ML model. Swab results and model risk scores were aggregated for each admission and assessed as predictors of HO-CDI. Holding sensitivity equal, we evaluated both approaches in terms of accuracy, specificity, and positive predictive value (PPV).

**Results:**

Of 2,044 admissions representing 1,859 patients, 39 (1.9%) developed HO-CDI. 23.1% (95% CI: 11.1–37.8%) of HO-CDI cases had at least one positive swab. At this sensitivity, model performance was significantly better than random but worse compared to swab surveillance—accuracy: 87.5% (86.0–88.9%) vs. 94.3% (93.3–95.3%), specificity: 88.7% (87.3–90.0%) vs. 95.7% (94.8–96.6%), PPV: 3.8% (1.6–6.4%) vs. 9.4% (4.3–16.1%). Combining swab AND model yielded lower sensitivity 2.6% (0.0–8.9%) compared to combining swab OR model at 43.6% (27.3–60.0%), and yielded PPV 7.1% (0.0–25.0%) vs. 43.6% (27.3–60.0%) respectively (**Figure 1**).

Figure 1. Surveillance & risk score performance.

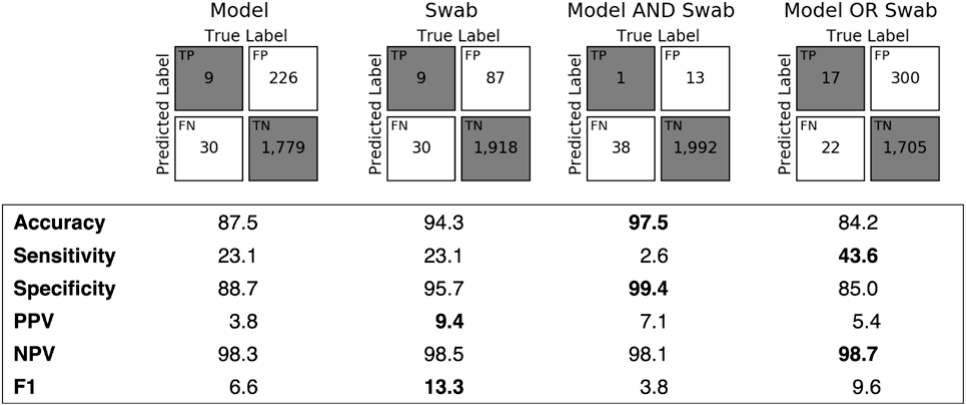

Binary classification performance metrics of ML model (Model), toxigenic C. difficile rectal swab surveillance (Swab), and combination approaches (Model AND Swab and Model OR Swab), reported in terms of percentage points. Bold numbers highlight the best performing approach for a given performance metric. The combined approach of monitoring the Model AND Swab yielded the highest accuracy 97.5% (95% confidence interval: 96.8%, 98.1%), it also had the highest specificity 99.4% (99.0%, 99.7%). The combined approach of monitoring the Model OR Swab yielded the highest sensitivity 43.6% (27.3%, 60.0%) and negative predictive value (NPV) 98.7% (98.2, 99.2%). Using the Swab alone yielded the highest PPV 9.4% (4.3%, 16.1%) and F1 score 13.3% (6.2%, 21.8%). These results highlight the complementarity of the model and swab-based approaches.

**Conclusion:**

Compared to swab surveillance using a ML model for predicting HO-CDI results in more false positives. The ML model provides daily risk scores and can be deployed using different thresholds. Thus, it can inform varied prevention strategies for different risk categories, without the need for resource intensive swabbing. Additionally, the approaches may be complimentary as the patients with HO-CDI identified by each approach differ.

**Disclosures:**

**Vincent B. Young, MD, PhD**, **American Society for Microbiology** (Other Financial or Material Support, Senior Editor for mSphere)**Vedanta Biosciences** (Consultant) **Krishna Rao, MD, MS**, **Bio-K+ International, Inc.** (Consultant)**Merck & Co., Inc.** (Grant/Research Support)**Roche Molecular Systems, Inc.** (Consultant)**Seres Therapeutics** (Consultant)

